# Modelling the joint accuracy of two diagnostic tests using evidence synthesis methods incorporating linked electronic health records

**DOI:** 10.23889/ijpds.v9i5.1652

**Published:** 2024-09-18

**Authors:** Leah Maizey, Josie Platcha, Tim Gammon, Matt Wray, Gavin Thompson, Laszlo Antal, Rosaland Archer

**Affiliations:** 1 Office for National Statistics

## Objectives

To compare the accuracy of different diagnostic pathways comprising of multiple diagnostic tests, statistical methods that evaluate combined or sequential test accuracy are required. This can only be achieved through joint synthesis of diagnostic accuracy data on all tests of interest, using models that account for dependencies between multiple tests. The aim of this research is to develop joint models to assess the diagnostic accuracy of two tests, using real-world evidence to inform within-study dependencies between tests.

## Approach

Using a case study in Alzheimer’s disease dementia (ADD), we will develop Bayesian meta-analysis models to evaluate the combined diagnostic accuracy of two tests. A novel application of trivariate copulas will be used to capture within-study dependencies between two tests assessed in the same patients. Individual participant data from Dementias Platform UK (DPUK) Data Portal – a repository of linked electronic health record data optimised for dementia research – will be used to estimate dependencies between diagnostic tests for ADD.

## Results

The DPUK Data Portal includes records of over 3 million people from 51 long-term cohort studies, including individual-level test results for ADD verified against confirmed diagnoses. Analysis will focus on modelling the diagnostic accuracy of different testing strategies for ADD, combining cognitive function, imaging, and biomarker tests.

## Conclusions and Implications

Results of this study have the potential to inform healthcare decision-making regarding optimal diagnostic pathways by enabling the comparison of different testing strategies. The incorporation of real-world data from the DPUK Data Portal makes better use of the available evidence.

